# Lymphepithelial carcinoma - a rare tumor of the larynx. Case Report

**DOI:** 10.3389/fsurg.2022.851481

**Published:** 2022-10-28

**Authors:** Piotr Nogal, Michalina Staśkiewicz, Joanna Jackowska, Małgorzata Wierzbicka

**Affiliations:** Department of Otolaryngology - Head and Neck Surgery, Poznan University of Medical Sciences, Poznań, Poland

**Keywords:** lymphoepithelial carcinoma (LEC), Schmincke's tumor, rare tumor location, laryngeal malignancy, laser microsurgery, head and neck surgery

## Abstract

Lymphoepithelioma was described in 1921 separately by Regaud and Schmincke as nests of non-keratinizing squamous cells embedded in a lymphoid stroma (Regaud) and isolated transitional cells scattered in lymphoid tissue resembling sarcoma (Schmincke). Lymphoepithelial tumors are the most common lesions of the nasopharynx, although they have also been reported in other localizations, such as the nasal cavity, maxillary sinus, the base of the tongue, parapharyngeal area, tonsils and thymus. Lymphoepithelioma of the larynx is extremely rare. We present a case of a 55-year-old patient treated due to this type of lesion to share our experience in the management of this type of malignancy and contribute to the field of rare laryngeal tumors diagnosis and treatment.

## Introduction

Lymphoepithelial carcinoma (LEC) is the most common lesions of the nasopharynx, although they have also been reported in other localizations, such as the nasal cavity, maxillary sinus, the base of the tongue, parapharyngeal area, tonsils and thymus (1). Lymphoepithelioma of the larynx is extremely rare with a 0.2% occurrence rate (1).

Compilation of LEC cases was performed by Faisal et al. with the distribution of the clinical and anatomic pathology variables in 46 patients with this type of tumor in the larynx and hypopharynx (2). Three more cases was described since this publication (Table 1).

**Table 1:** New cases described after publication of Faisal et al. (2).

No.	Age:	Sex:	Presentation:	Site:	TNM:	Tumor spread:	Treatment:	Follow-up acc. to publication	Reccurency	Literature:
1.	60	M	Chronic laryngitis	Supraglottis and Hypopharynx	No data	No data	Chemoradiotherapy	3 years	*no*	(3)
2.	70	M	Hoarseness, dysphagia	Supraglottis	T3 N2c M0	No	Pharyngolaryngectomy and bilateral selective neck dissection, adjuvant radiotherapy	2 years	*no*	(4)
3.	70	M	Hoarseness, neck tumor	Glottis	T3 N3b M1	Mediastinum	Chemotherapy, secondary laryngectomy and selective neck dissection	8 months	*no*	(5)

We present a case of a 55-year-old patient with Schmincke's tumor of the larynx.

## Case report

**Figure 1:**
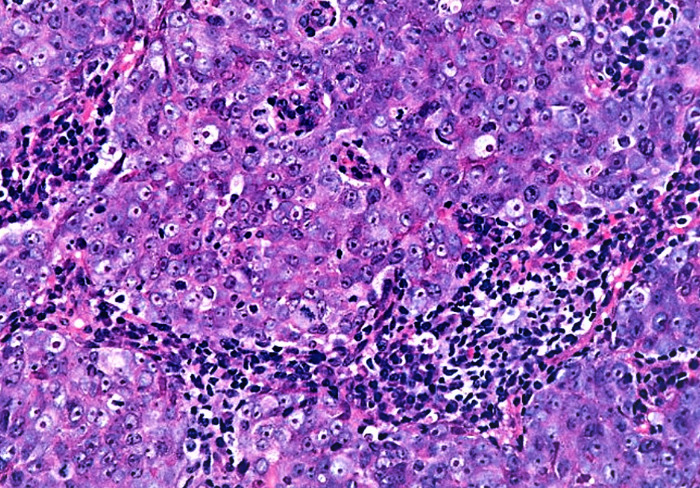
Undifferentiated carcinoma- sheet of tumor cells with few intermixed lymphocytes (H + E stain).

**Figure 2:**
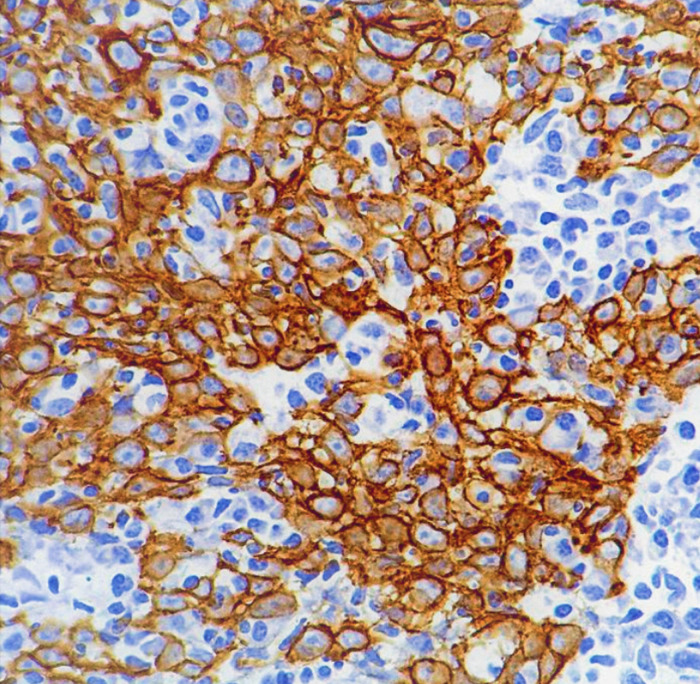
Histopathological examination showing positivity for cytokeratin.

A 55-year-old caucasian male patient was admitted to the laryngology ward due to the suspicion of a tumor involving the larynx. The patient had hoarseness lasting for 2 months prior to the admision, without other symptoms. He was a heavy smoker (>20 cigarettes almost over 30 years), suffering from hypertension and psoriasis. The laryngological examination revealed the smooth tumor of the left side of the larynx involving the left vocal fold, left vestibular fold and the petiole of the epiglottis. The surgical procedures were scheduled- the patient underwent two microlaryngoscopies with the sampling of the tumor tissue resulting with the diagnosis of malignant lesion, then the patient was referred to transoral laser-microsurgical resection (Table 2). Final histopathological examination revealed nonkeratinizing undifferentiated carcinoma, Schmincke pattern (Figures 1, 2).

**Table 2:** Procedures and histopathological examination results.

Date:	Procedure:	Histopathological result:
17.01.2020	Micorlaryngoscopy (cold steel sampling)	High-grade dysplasia of the epithelium
14.02.2020	Micorlaryngoscopy (cold steel sampling)	Neuroendocrine carcinoma suspicion; final result: nasopharyngeal carcinoma, nonkeratinizing undifferentiated carcinoma, Schmincke pattern
17.03.2020	Micorlaryngoscopy (laser CO2 resection)	Nasopharyngeal carcinoma, nonkeratinizing undifferentiated carcinoma, Schmincke pattern; the histopathological examination revealed the immunochemistry markers in tumor cells: p16 -, CKPAN +, p63 +, p40 -, LCA -, Ki67 in 80% of cells, CD56 +, synaptophysin -, chromogranin A -, TTF1 -, CK7 +

**Figure 3:**
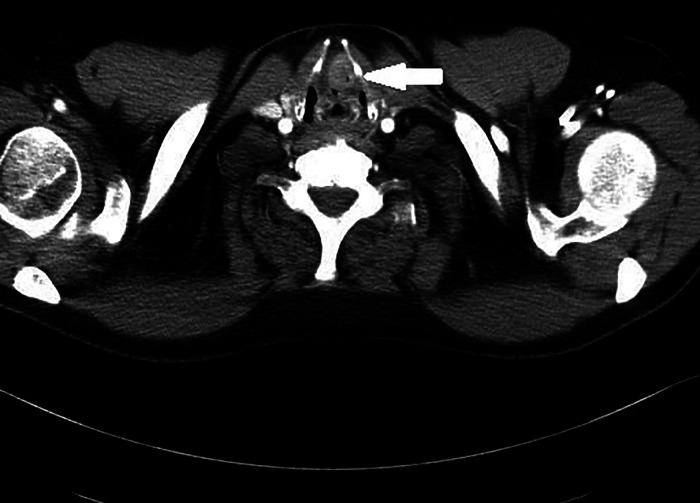
Pathological mass located in the left vestibular fold (lesion marked with an →).

**Figure 4:**
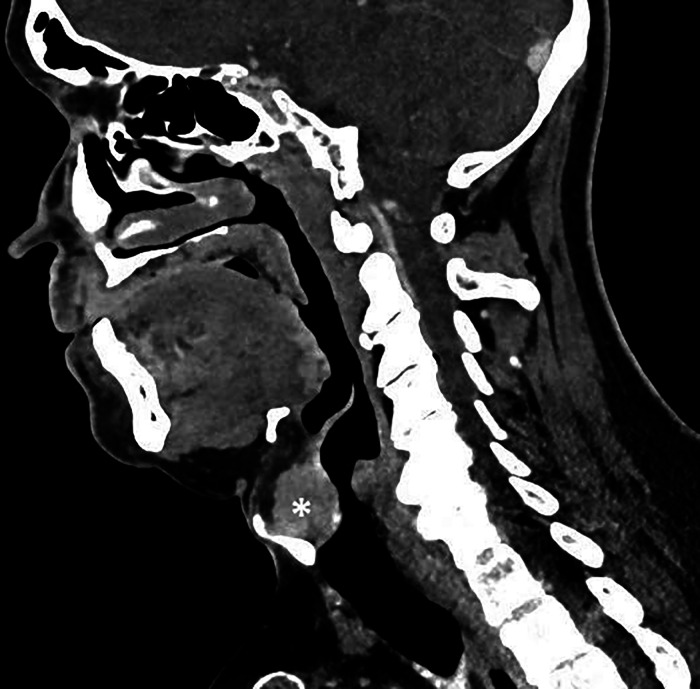
The entrance to the larynx, epiglottis, subglottic area of the larynx and the part of the trachea included in the examination (lesion marked with *).

The computed tomography (CT) scans made before surgical resection with the use of CO2-laser showed the presence of a tumor measuring 19 × 12 mm and height 21 mm, within the pedicle of the epiglottis on the left side and the anterior 2/3 of the left vestibular fold, invading the anterior commissure and reaching the vocal fold without any obvious features of vocal fold infiltration. In addition, the correct CT image of the entrance to the larynx, epiglottis, subglottic area of the larynx and the part of the trachea included in the examination (Figure 3, 4).

After the surgical resection the Oncological Board referred the patient for adjuvant radiotherapy. He received a full cycle of radiotherapy as planned- 60 Gy. The patient stays under the care of an outpatient clinic. No signs of tumor recurrence were observed in over 18 months of observation. The patient has a socially efficient voice, but he's dealing with the side-effects after radiotherapy (oral mucositis, skin irritation on the neck) on a manageable level.

## Discussion

Lymphoepithelioma was described in 1921 separately by Regaud and Schmincke as nests of non-keratinizing squamous cells embedded in a lymphoid stroma (Regaud) and isolated transitional cells scattered in lymphoid tissue resembling sarcoma (Schmincke) (6, 7).

The non-differentiated tumor (called Schmincke's tumor) is more prevalent in the African and Chinese populations (2, 6). There are two peaks of incidence of the tumor according to age: between 20 and 30 years, and after 60 years of age (1, 6). According to the research of Faisal et. all. the mean age of presentation is 64 years (range 40–82 years) with predominant presentation in males. The most commonly involved subsites are the supraglottis and hypopharynx. Smoking and alcohol are major contributing factors in the LEC development (2).

The tumor is rarely located outside of the nasopharynx. In 75% of cases lymph node invasion at the time of diagnosis is present and metastatic disease is described in almost 30% of the cases. The most common sites of metastasis are bones, lungs, and liver (8, 9). No signs of cervical lymphadenopathy or metastatic disease were observed in our patient's case. The association with some HLA haplotypes (A2, A33, B46, B58) was noted (9). Dermatomyositis often manifests as a paraneoplastic syndrome (9). However, most common complaints are hoarseness (⅓ of patients), dysphagia (⅕ of patients) and cervical mass (⅕ of patients) (2).

The lymphoepithelioma of the larynx may arise from lymphatic tissue in the laryngeal ventricle. The microscopic studies on the origin and biology of the tumor showed the presence of single or double laryngocele in 70% of cases of laryngeal and parapharyngeal carcinomas in surgical specimens (9, 10). Cylindrical or squamous epithelium with lymphoid tissue resembling the typical histology of lymph nodes and lymphoid tissue of Waldeyer's ring was observed during the laryngoceles evaluation. This lymphoepithelial tissue has been proposed to be a tonsil of the larynx. Lymphoepithelioma may arise from these structures. Active basal epithelium in the larynx, similar to the epithelium found in the tonsillar crypts, is also proposed to be alternatively the place of origin of these lesions. Lymphocytes are believed to be a nonneoplastic component of the tumor since only the epithelial component is found in the metastatic tissue (1, 8–10).

The lymphomas and lymphoepitheliomas of the larynx are very difficult to distinguish clinically, usually presenting as smooth supraglottic masses involving the epiglottic area or aryepiglottic fold (Table 3). Cytokeratin immunohistochemistry is helpfull in highlighting the irregular infiltrative nature of lymphepithelial carciona. Malignant differential diagnoses that need to be distinguished from these type of tumor includes lymphoma and melanoma. In our patient's case the tumor was a smooth lesion involving the supraglottic region. The lymphoepithelioma of the larynx shares many similarities with those of the nasopharynx: the significant metastatic potential to the mediastinum, lung, and abdomen and high radiosensitivity (1).

**Table 3:** Differential histopathological diagnosis.

Type of the tumor	Description
Lymphoepithelial carcinoma- Undifferentiated/Schmincke's tumor	• Lesion is microscopically characterized by tumor cells with spindle-to-oval hyperchromatic or vesicular nuclei bearing prominent nucleoli, which feature scattered mitotic activity; • Variable numbers of intermixed lymphocytes and plasma cells; Two classically described tumor growth patterns: – The “Regaud” pattern with a solid sheet-like tumor cell growth pattern, – The “Schmincke” presenting separated or loosely attached tumor cells (described as a reticulated pattern) with prominent intermixed lymphocytes • Cytokeratin-positive cells Stick in groups or in a reticulated pattern mixed with lymphocytes
Lymphoma	• Tumor can be difficult to differentiate from carcinoma on morphological features alone; • Tumor cells form cohesive groups and have ill-defined cell borders; • An immunohistochemistry panel (LCA and cytokeratin) has great value in the diagnosis
Melanoma	• Three histological types: epithelioid, spindled, or undifferentiated; • Presence of melanin pigment; • Multinucleate lesion; • Giant cells present; • Positivity for S-100, HMB-45, melan-A

Due to the rarity of LEC in the larynx and hypopharynx the current literature provides only recommendations and there are no available treatment guidelines.Treatment options include surgery and radiotherapy (primary and adjuvant) (9). Due to the radiosensitivity of the lesion, radiotherapy should be considered as a first-line-therapy providing good local control (11–13). In this case the patient prefered surgical resection followed by the adjuvant treatment. This course of treatment is recommended in advanced cases. Adjuvant chemotherapy may be useful (10, 11). Neoadjuvant or accompanying chemotherapy for lymphoepithelial carcinoma may be recommended in case of early regional adenopathy, which may decrease the risk of metastatic disease development (9–14). The chemotherapy protocol in advanced cases of LEC suggested by Bugada et. all is EXTREME regimen therapy (cisplatin, cetuximab and fluorouracil) (5). This protocol is recommended as the standard care for recurrent/metastatic head and neck cancer not eligible for surgery or chemotherapy with curative intent, according to NCCN guidelines (2022). The standard protocol of therapy consist of: (1) Cetuximab 400 mg/mEq as first administration and then 250 mg/mEq on days 1, 8, 15 and then 21; (2) Cisplatin 100 mg/mEq in single administration, repeated on day 21 or as an alternative may 20 mg/mEq for 5 days and then repeated on 21 day; cisplatin dosage should be modified according to age, general condition of the patient and his renal function; the change for carboplatin is possible; (3) Fluorouracil (5-FU) 200 mg/mEq on days 1, 2, 3, 4, 5 with the repetition after 21 days. Radiotherapy protocols vary according to stage of the tumor, usually with the 70 Gy (2.0 Gy/fraction) per 7 weeks and for the site of suspected subclinical spread: 44–50 Gy (2.0 Gy/fraction) to 54–63 Gy (1.6–1.8 Gy/fraction). This protocol is suggested in NCCN guidelines (2022) in the treatment of advanced head and neck cancer (3, 5).

Our patient received a full cycle of planned adjuvant radiotherapy. Chemotherapy was not scheduled in our patient's case.

Concluding, we have presented a case of a rare laryngeal malignancy treated with success by the surgical excision and adjuvant radiotherapy.

## Data Availability

The raw data supporting the conclusions of this article will be made available by the authors, without undue reservation.
